# Carboplatin plus nab‐paclitaxel for recurrent small cell lung cancer: A phase II study

**DOI:** 10.1111/1759-7714.14394

**Published:** 2022-03-22

**Authors:** Naoya Ikeda, Ryo Arai, Sayo Soda, Takashi Inoue, Nobuhiko Uchida, Yusuke Nakamura, Meitetsu Masawa, Yoshitomo Kushima, Hiroaki Okutomi, Akihiro Takemasa, Yasuo Shimizu, Seiji Niho

**Affiliations:** ^1^ Department of Pulmonary and Clinical Immunology Dokkyo Medical University School of Medicine Tochigi Japan; ^2^ Department of Respiratory Medicine Sano Kosei General Hospital Tochigi Japan

**Keywords:** carboplatin, interstitial pneumonia, nab‐paclitaxel, small cell lung cancer

## Abstract

**Background:**

We conducted a phase II study of carboplatin plus nab‐paclitaxel for the treatment of small cell lung cancer (SCLC) after the failure of a prior standard chemotherapy containing platinum, etoposide, irinotecan, and amrubicin if indicated. Patients with interstitial pneumonia complications were included in the study.

**Methods:**

Patients received 100 mg/m^2^ of nab‐paclitaxel weekly (on days 1, 8, and 15) and an AUC 5 of carboplatin on day 1. The study treatment was repeated every 3 weeks until disease progression or the appearance of unacceptable toxicities. The primary endpoint was the objective response rate.

**Results:**

A total of 21 patients were enrolled, all of whom were eligible for inclusion in the analysis. Twelve patients had pre‐existing interstitial pneumonia. The overall response rate was 19.0% (90% confidence interval [CI]: 6.8%–38.4%). The lower limit of the 90% CI for the response rate did not exceed the prespecified threshold value of 10%. Among the 12 patients with pre‐existing interstitial pneumonia, the response rate was 25%. The median progression‐free survival time was 2.5 months (95% CI: 1.5–3.4 months), and the median survival time was 5.1 months (95% CI: 2.1–8.1 months). Two patients developed interstitial lung disease; both of these patients had pre‐existing interstitial pneumonia. One of the patients died from interstitial lung disease.

**Conclusion:**

Combination chemotherapy with carboplatin plus nab‐paclitaxel for recurrent SCLC had a modest activity, although the primary study endpoint was not met. Further investigation of this regimen for patients with recurrent SCLC and interstitial pneumonia is warranted.

## Graphical abstract

We conducted a phase II study of carboplatin plus nab‐paclitaxel for the treatment of small cell lung cancer after the failure of a prior standard chemotherapy containing platinum, etoposide, irinotecan, and amrubicin if indicated. Patients with interstitial pneumonia complications were permitted. Combination chemotherapy with carboplatin plus nab‐paclitaxel for recurrent small cell lung cancer had a modest activity with manageable toxicities, although the primary study endpoint was not met.

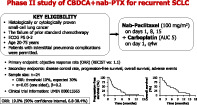

## INTRODUCTION

Lung cancer is a leading cause of cancer‐related death worldwide. Small cell lung cancer (SCLC) accounts for approximately 15% of all forms of lung cancer. SCLC is a high‐grade neuroendocrine tumor of the lung and is characterized by rapid growth and wide metastasis. Sixty percent or more of patients are stage IV at diagnosis.^1^ The mainstay of treatment for extensive‐stage (ES) SCLC is systemic chemotherapy. A Japanese randomized phase III study demonstrated that cisplatin plus irinotecan had a significant survival benefit, compared with cisplatin plus etoposide, in patients with ES‐SCLC (JCOG 9511).^2^ In contrast, similar clinical trials conducted in western countries failed to show a survival benefit for combination chemotherapy with cisplatin plus irinotecan.^3^, ^4^, ^5^ Therefore, platinum plus etoposide combination chemotherapy has been recognized as a standard first‐line regimen globally. Recently, two randomized phase III studies showed survival prolongation by the addition of a PD‐L1 inhibitor to platinum plus etoposide.^6^, ^7^ Atezolizumab or durvalumab combined with platinum plus etoposide has become a new standard treatment for patients with ES‐SCLC. However, the 1‐year progression‐free survival (PFS) rate was 13%–18%, and 90% of patients ultimately experience progression.^8^

Rechallenge with platinum plus etoposide (especially in patients with sensitive relapses), amrubicin, topotecan, irinotecan and so on can be used as a second‐line or later chemotherapy.^9^, ^10^, ^11^, ^12^, ^13^, ^14^, ^15^ The response rate ranges from 6.4% to 47% for these treatments. A cohort study conducted in Germany demonstrated that 50% and 22% of patients received second‐ and third‐line treatments for ES‐SCLC.^16^ Effective chemotherapy for recurrent SCLC after the above mentioned chemotherapeutic regimens is limited, and the development of further effective drugs is necessary. According to previous trials, monotherapy with paclitaxel (PTX) or combined therapy with PTX plus carboplatin (CBDCA) for recurrent SCLC produced response rates of 25%–29%.^17^, ^18^ PTX can cause adverse events such as hypersensitivity reactions and peripheral neuropathy, which sometimes lead to treatment termination. Nab‐PTX is a nanoparticle form of albumin‐bound PTX that exhibits effective intratumoral accumulation, shortens the infusion time, and is expected to reduce the risk of hypersensitivity reaction. A relatively low dose and weekly administration of nab‐PTX might also reduce peripheral neuropathy and febrile neutropenia.^19^, ^20^, ^21^ Based on these rationales, we conducted a phase II study of CBDCA plus nab‐PTX in patients with recurrent SCLC after standard chemotherapy.

## METHODS

### Patient population

Patients were required to have histological‐ or cytologically‐confirmed SCLC and the failure of a prior standard chemotherapy containing platinum, etoposide, irinotecan, or amrubicin if indicated. Other criteria were: patients age 20 years or more and less than 76 years; an ECOG performance status (PS) of 0–2; measurable disease; adequate organ function (i.e., neutrophil count ≥1500/mm^3^, platelet ≥100 000/mm^3^, hemoglobin ≥9.0 g/dl, AST and ALT ≤2.5 times the upper limit of the reference range, total bilirubin ≤1.5 mg/dl, creatinine ≤1.5 mg/dl); no abnormal findings suggesting clinical problems on an electrocardiogram; no peripheral neuropathy of grade 2 or worse; no symptomatic brain metastasis; no pregnancy or breast‐feeding; no active concomitant malignancy; no radiotherapy within the past 4 weeks; no uncontrolled angina; no cardiac infarction within 3 months; no uncontrolled diabetes mellitus; no liver cirrhosis; no pleural and/or pericardial effusion requiring drainage; and no allergy to nab‐PTX or CBDCA. Patients with interstitial pneumonia (IP) were permitted. Diagnosis of IP was based on the radiological findings. Eligibility criteria did not include lower limit value of SpO_2_ or PaO_2_. All patients were required to provide written informed consent, and the institutional review board approved the protocol (no. 26063 at Dokkyo Medical University Hospital).

### Treatment plan

Patients received 100 mg/m^2^ of nab‐PTX weekly (on days 1, 8, and 15) and an AUC 5 of CBDCA on day 1. The study treatment was repeated every 3 weeks unless there was evidence of disease progression or the appearance of unacceptable toxicities. Administration of granulocyte colony‐stimulating factor was allowed if a neutrophil count of ≤500/mm^3^, or fever over 38°C with a neutrophil count of ≤1000/mm^3^ was observed.

### Evaluation

Computed tomography (CT) of the chest and abdomen was performed every 6 weeks. Magnetic resonance imaging (MRI) or a CT scan of the brain was also performed if patients had brain metastases. The response was assessed by the investigator per RECIST version 1.1.^22^ Toxicity was graded according to the Common Terminology Criteria for Adverse Events (CTCAE), version 4.0.

### Endpoint

**FIGURE 1:**
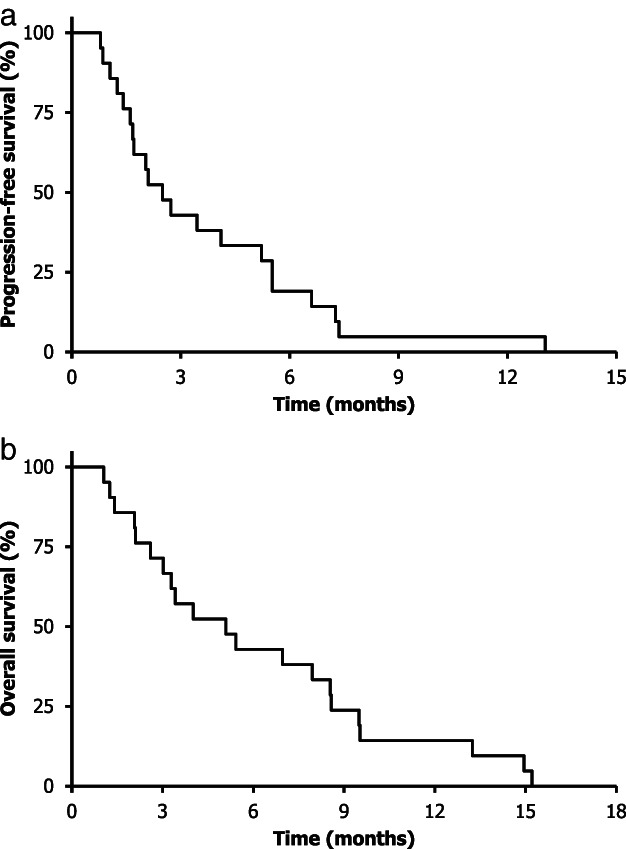
Progression‐free survival (a) and overall survival (b) for the entire study population

The primary endpoint was the objective response rate, and the secondary endpoints were the disease control rate, PFS, overall survival (OS), and adverse events. PFS was defined as the interval between enrollment in this study and the first documented evidence of disease progression or death, whichever occurred first. OS was defined as the interval between enrollment in this study and death or the final follow‐up visit. Survival was estimated using the Kaplan–Meier method (Figure 1).

### Statistical analysis

The sample size was determined to be 24, with a one‐sided alpha of 0.05, a beta of 0.2, and expected and threshold values for the primary endpoints of 30% and 10%. A previous phase II study of CBDCA plus PTX for refractory SCLC demonstrated that the response rate was 25% (95% confidence interval [CI]: 10%–40%).^18^ The use of nab‐PTX can reduce the administration time and the risk of hypersensitivity reaction and peripheral neuropathy, compared with PTX.^23^ Therefore, the threshold was set as 10%, which was identical to the lower limit of the 95% CI of the response rate in the above‐mentioned study examining CBDCA plus PTX.

This study was designed as a multicenter, prospective phase II study and was registered with the UMIN Clinical Trials Registry (number UMIN 000015565).

## RESULTS

### Patient population

A total of 21 patients were enrolled in this study between September 2014 and September 2019 from two institutions (Table 1). Patient accrual was delayed because the condition of most patients had deteriorated with disease progression after standard chemotherapy and they were ineligible for study enrollment. Due to slow enrollment, accrual was stopped before the planned sample size was achieved. All the patients were eligible for inclusion in the analysis. The median age was 70 years old (range: 54–85), 18 patients were men (85.7%), 20 patients had extensive disease at study entry, and the median duration between the last administration of the previous treatment and relapse was 22 days (range, 7–84 days); therefore, all the patients had refractory relapse. Twelve patients (57.1%) had IP complications. All but one patient had IP at the diagnosis of SCLC. The remaining one patient developed drug‐induced IP during the prior chemotherapy of topotecan. Four and eight patients had usual interstitial pneumonia (UIP) and non‐UIP patterns, respectively. No patients required oxygen therapy at the time of enrollment. Six patients (28.6%) had brain metastases. Nine patients had received one prior treatment regimen; eight of these patients had IP complications. Of the remaining four patients with IP, three patients had received two prior treatment regimens, and one patient had received eight prior treatment regimens that included four rechallenge treatments with CBDCA plus ETP. Median number of prior chemotherapy regimens in patients with or without pre‐existing IP was one and two, respectively.

**TABLE 1:** Patient characteristics (*n* = 21)

Characteristics			*N*	%
Age (years)	Median (range)		70 (54–85)	
Gender	Male		18	85.7
	Female		3	14.3
ECOG performance status	0		13	61.9
	1		1	4.8
	2		7	33.3
Tumor stage	Limited		1	4.8
	Extensive		20	95.2
Metastatic site	Brain		6	28.6
	Bone		6	28.6
	Liver		4	19.0
Interstitial pneumonia	No		9	42.9
	Yes		12	57.1
	Radiological features of interstitial pneumonia	UIP	4	19.0
		Non‐UIP	8	38.1
Prior treatment	Chemotherapy		20	95.2
	Chemoradiotherapy		1	4.8
Prior chemotherapy regimen (multiple choices)	Platinum plus etoposide		19	90.5
	Platinum plus irinotecan		9	42.9
	Amrubicin		7	33.3
	Others		2	9.5
Number of prior chemotherapy regimens	1		9	42.3
	2		7	33.3
	3		4	19.0
	8		1	4.8

Abbreviations: ECOG, Eastern Cooperative Oncology Group; UIP, usual interstitial pneumonia.

### Treatment compliance

The median number of cycles was three (range: 1–10), and the median number of nab‐PTX administrations was six (range, 2–27). The dose intensity was 66.5% for CBDCA and 66.7% for nab‐PTX. Seventeen patients (90.0%) experienced a treatment delay of the next cycle lasting more than 1 week, and 11 patients (52.4%) skipped a nab‐PTX treatment (all of which would have occurred on day 15).

### Efficacy

Four and six patients achieved partial responses and stable disease, respectively (Table 2). An imaging evaluation was not conducted in one patient with a brain metastasis (protocol deviation), and the response was assessed as not evaluable in this patient. The objective response rate was 19.0% (90% CI: 6.8%–38.4%; 95% CI: 5.4%–41.9%), and the disease control rate was 47.6% (95% CI: 25.7%–70.2%). The primary endpoint was not met, since the lower limit of the 90% CI for the response rate was lower than 10% of the threshold.

**TABLE 2:** Response

Response	Number of patients	%
CR	0	0
PR	4	19.0
SD	6	28.6
PD	10	47.6
NE	1	4.8

Abbreviations: CR, complete response; NE, not evaluable; PD, progressive disease; PR, partial response; SD, stable disease.

Among the 12 patients with IP, three patients had partial responses, four had stable diseases, and five had progressive diseases; the response rate was 25% (95% CI: 5.4%–57.2%). Among the nine patients who did not have IP, one patient had a partial response, two had stable diseases, four had progressive diseases, and one patient was not evaluable; the response rate was 11.1% (95% CI: 2.8%–48.2%). Among the nine patients with one prior treatment regimen, the response rate was 22.2%; among seven patients with two prior‐treatment regimens, the response rate was 28.6%; and among five patients with three or more prior‐treatment regimens, there were no responders.

PFS and/or OS events were observed in all 21 patients. The median PFS was 2.5 months (95% CI: 1.5–3.4 months), and the median survival time was 5.1 months (95% CI: 2.1–8.1 months).

### Safety

Grade 3 or 4 hematological toxicity was observed in 18 patients (85.7%). Eleven patients (52.4%) experienced grade 3 or 4 neutropenia. The most common toxicity regardless of the grade was anemia (*n* = 20, 95.2%). Grade 3 or 4 nonhematological toxicity was observed in seven patients (33.3%) (Table 3). Interstitial lung disease (ILD) did not occur in the nine patients who did not have IP prior to treatment. On the other hand, among the 12 patients with pre‐existing IP, one of the four patients with a UIP pattern and one of the eight patients with a non‐UIP pattern experienced acute ILD. The former patient developed grade 2 ILD, and the latter patient died. This was the only treatment‐related death in this series (4.8%).

**TABLE 3:** Treatment‐related adverse events (CTCAE v4.0)

Adverse event	All grade		Grade 3		Grade 4	
	*n*	%	*n*	%	*n*	%
Hematological adverse events						
Leukocytopenia	19	90.5	9	42.9	0	0
Neutropenia	18	85.7	8	38.1	3	14.3
Anemia	20	95.2	5	23.8	0	0
Thrombocytopenia	18	85.7	4	19	1	4.8
Febrile neutropenia	1	4.8	0	0	0	0
Blood bilirubin increased	4	19	1	4.8	0	0
Aspartate aminotransferase increased	9	42.9	2	9.5	0	0
Alanine aminotransferase increased	8	38.1	1	4.8	0	0
Creatinine increased	5	23.8	0	0	0	0
Nonhematological adverse events						
Anorexia	11	52.4	4	19	0	0
Nausea	9	42.9	1	4.8	0	0
Vomiting	1	4.8	0	0	0	0
Fatigue	12	57.1	0	0	0	0
Constipation	8	38.1	0	0	0	0
Diarrhea	2	9.5	0	0	0	0
Hiccups	1	4.8	0	0	0	0
Peripheral sensory neuropathy	3	14.3	1	4.8	0	0
Mucositis oral	1	4.8	0	0	0	0
Pneumonitis	2	9.5	0	0	1	4.8

Abbreviation: CTCAE, Common Terminology Criteria for Adverse Events.

Treatment was discontinued because of adverse events in four patients: acute ILD (*n* = 2), peripheral neuropathy (*n* = 1), and complications from myelodysplastic syndrome (*n* = 1).

## DISCUSSION

This prospective study examined CBDCA plus nab‐PTX for the treatment of recurrent SCLC. The response rate was 19.0% (90% CI: 6.8%–38.4%; 95% CI: 5.4%–41.9%), and the primary study endpoint was not met. A previous phase II study of CBDCA + PTX for recurrent SCLC as a second‐line treatment had a response rate of 25% (95% CI: 10%–40%).^18^ In our study, 57.1% of the patients had received two or more lines of prior treatment, and all the patients had shown progression within 90 days from the end of the prior treatment; in other words, these patients had refractory disease, and this might have resulted in the lower‐than‐expected response rate. Another previous phase II study of nab‐PTX as a single agent for the second‐line treatment of recurrent SCLC had a response rate of 16.0% (90% CI: 6.1%–33.5%), a median OS of 3.65 months (95% CI: 2.07–4.57 months), and a median PFS of 1.84 months (95% CI: 1.02–3.16 months).^24^ Thus, combination chemotherapy with CBDCA plus nab‐PTX might be more efficacious than single agent nab‐PTX. A Japanese phase II study of amrubicin for the treatment of refractory SCLC resulted in a response rate of 32.9% (95% CI: 22.9%–44.2%) and a median OS of 8.9 months.^10^ Amrubicin monotherapy might be an effective treatment option for refractory SCLC; however, amrubicin is not recommended for patients with IP because a retrospective analysis of amrubicin for SCLC reported that 7% of patients developed acute ILD. In particular, the incidence of acute ILD was 33% (4 out of 12) among patients with pre‐existing IP.^25^ In addition, previous phase II studies of irinotecan in patients with refractory SCLC and previously untreated NSCLC demonstrated that the incidence of ILD was 8%–13%.^15^, ^26^ Therefore, irinotecan is also not recommended for patients with IP.

The response rate in patients with or without IP was 25% and 11.1%, respectively. Because of limited treatment options for patients with IP, the median number of prior lines of chemotherapy in patients with or without IP was one and two, respectively. A small number of prior treatment lines in patients with IP probably resulted in a better response than those without.

The majority of patients with SCLC were smokers, and 10%–20% of patients had IP at the time of the diagnosis of SCLC.^27^, ^28^, ^29^ Two of the 12 patients with pre‐existing IP developed acute ILD in our study, leading to one treatment‐related death. In the remaining 10 patients, CBDCA and nab‐PTX were safely administered without the development of acute ILD. One of the four patients with a UIP pattern and one of the eight patients with a non‐UIP developed acute ILD. The frequency between the two groups was not statistically different; however, the sample size was too small to evaluate which IP pattern might be associated with a risk of ILD. A combination of chemotherapy with platinum plus PTX or nab‐PTX is frequently used in patients with non‐small cell lung cancer and IP. The incidence of acute ILD in patients treated with these regimens is reportedly 4.3%–9%.^30^, ^31^, ^32^ The administration of amrubicin or irinotecan is associated with a high risk of ILD in patients with pre‐existing IP^33^; therefore, treatment options are limited in patients with advanced SCLC and IP. A previous retrospective study of PTX, nab‐PTX, or CBDCA plus PTX treatment in patients with recurrent SCLC and IP demonstrated that five of 17 patients (29.4%) developed ILD.^34^ In another retrospective study of PTX, CBDCA plus PTX, or topotecan treatment as second‐line treatments in patients with SCLC and IP, three of 23 patients (13%) developed grade 3 or higher ILD; all three of these patients had a UIP pattern.^35^ A retrospective study in 109 patients with advanced lung cancer and IP indicated that the incidence of ILD was significantly higher in patients with a UIP pattern on CT findings, compared with those with a non‐UIP pattern.^36^ Therefore, the risk of ILD might be elevated in patients with SCLC and a UIP pattern who are treated with nab‐PTX.

A previous phase II study of CBDCA plus PTX for recurrent SCLC demonstrated that the incidence of grade 3 or 4 neutropenia was 37%.^18^ Although 11 patients (52.4%) experienced grade 3 or 4 neutropenia in our study, none of the patients developed neutropenic fever. If patients develop myelosuppression during treatment, the administration of nab‐PTX could be skipped on day 8 or 15. The weekly administration of nab‐PTX might contribute to the avoidance of febrile neutropenia.

The present study had some limitations. First, because of the small sample size, a subset analysis based on the presence or absence of IP was not possible. Second, as a blinded independent central review of the imaging results was not conducted, the response evaluation might not be accurate.

In conclusion, our study did not meet the primary study endpoint; however, combination chemotherapy with CBDCA plus nab‐PTX seems to be safe in patients with IP. Further investigation of this regimen for patients with recurrent SCLC and IP is warranted.

## CONFLICT OF INTEREST

S. N. reported grants and personal fees from Taiho Pharmaceutical during the conduct of the study; grants and personal fees from Eli Lilly Japan, AstraZeneca, Pfizer, Boehringer Ingelheim, MSD, Chugai Pharma, Ono Pharmaceutical, Takeda, Shionogi, grants from Sanofi, Merck Serono, Teijin Pharma, personal fees from Bristol Myers Squibb, Nippon Kayaku, Yakult, Kyorin, Janssen, Novartis, outside the submitted work. All other authors have no conflicts of interest.
